# Referring patients with stable moderate-to-advanced chronic kidney disease back to primary care: a feasibility study

**DOI:** 10.3399/BJGPO.2021.0177

**Published:** 2022-05-04

**Authors:** Carola van Dipten, Wim de Grauw, Marc ten Dam, Willem Assendelft, Nynke Scherpbier-de Haan, Jack Wetzels

**Affiliations:** 1 Department of Primary and Community Care, Radboud Institute for Health Sciences, Radboud University Medical Center, Nijmegen, The Netherlands; 2 Department of Internal Medicine, Canisius Wilhelmina Hospital, Nijmegen, The Netherlands; 3 Department of General Practice and Elderly Care Medicine, University of Groningen, University Medical Centre Groningen, Groningen, The Netherlands; 4 Department of Nephrology, Radboud Institute for Health Sciences, Radboud University Medical Center, Nijmegen, The Netherlands

**Keywords:** renal insufficiency, chronic, back referral, primary health care, patient participation, feasibility studies

## Abstract

**Background:**

Care for patients with chronic kidney disease (CKD) necessitates tailored pathways between primary and secondary care. It is unknown if back referring patients with CKD is safe and effective.

**Aim:**

To study the feasibility of discharging patients with stable moderate-to-advanced CKD from secondary to primary care, and to evaluate quality of care (QoC) and patients’ and GPs‘ experiences.

**Design & setting:**

A monocentre prospective mixed-method study in the Netherlands.

**Method:**

Patients were included who met pre-determined back-referral (BR) criteria. Patients were discharged with personalised information guides and transfer letters. GPs had the option of consulting a nephrologist by telenephrology. Renal outcomes, QoC, and experiences were collected after 1 year.

**Results:**

Eighteen patients were included. The mean age was 73 years; the mean estimated glomerular filtration rate (eGFR) was 33.2 ml/min/1.73 m^2^ at baseline. After 1 year, four patients had received either no or incomplete monitoring, and one patients’ blood pressure was too high. The remaining 13 had stable eGFR, proteinuria, and metabolic parameters. Patients were satisfied with information provision and treatment by GPs but expected more frequent monitoring. In one-third of cases, monitoring frequency was decreased by GPs for several reasons. GPs believed they had sufficient knowledge to treat patients with CKD, but indicated they needed support besides a transfer letter.

**Conclusion:**

BR seems safe and feasible for patients with stable moderate-to-advanced CKD who meet specific criteria. Patients have good renal outcomes after 1 year and are satisfied with treatment. GP QoC can be improved, particularly completeness and monitoring frequency.

## How this fits in

Rising incidence in CKD necessitates optimal use of primary and secondary care facilities; for example, BR to primary care in case of non-progressive kidney disease. It is unknown what factors help facilitate an effective and safe transition from secondary to primary care for patients with stable moderate-to-advanced CKD. Therefore, patients with CKD were referred back to primary care, and QoC, renal outcomes, and patients’ and GPs’ experiences after 1 year were studied.

## Introduction

CKD contributes to cardiovascular morbidity and mortality.^1^ CKD prevalence is increasing owing to population ageing and the rising incidence of hypertension and diabetes.^2^ Consequently, care for patients with CKD necessitates tailored pathways between primary and secondary care,^3^ and the optimal use of primary and secondary care facilities. Management in primary care requires a balance between timely consultations and referral of patients with uncontrolled or progressive diseases,^4^ and BR to primary care when possible.

Several international CKD guidelines provide GPs with tools for determining whether referral to secondary care is needed.^5–7^ However, there is no description of the best circumstances for referring patients back to primary care. Therefore, BR criteria were developed in a regional consensus meeting and the records of patients under nephrologist care were reviewed. It was concluded that one-quarter of the patients under nephrologist care could be referred back to primary care.^8^ However, BR depends on more factors than clinical suitability alone. Involved patient and professional perspectives, conditions for collaboration, and maintaining quality of care are equally important.

Information on patients’ preferences for care setting is scarce. One study indicated patients prefer care by GPs with access to nephrologists.^9^ GPs stated that, besides conservative care for older and frail patients with advanced CKD, they had little experience in treating younger patients with advanced CKD and needed guidance from nephrologists.^10^ It was found nephrologists in Germany collaborated with GPs.^11^ Both GPs and nephrologists were willing to collaborate. They prefered shared care,^9,12^ provided adequate information exchange was guaranteed.^13^ Several older studies indicated discharge to primary care was generally safe.^14–16^

In hospitals and general practices, it is important to involve patients as much as possible because CKD requires an optimal lifestyle, as well as for managing symptoms and treatment and activating resources.^17^ Well-instructed patients can also monitor QoC for their CKD after being referred back to primary care.

It is still unknown what factors facilitate effective and safe transitions from secondary to primary care for patients with moderate-to-advanced non-progressive kidney disease. Accordingly, the feasibility of BR was studied by evaluating QoC for patients with CKD who were discharged back to primary care, including patient participation in follow-up care, and the experiences of patients and GPs in the process of BR and transition of care.

## Method

### Study design

The prospective study started in the Canisius Wilhelmina Hospital (CWZ) in the Netherlands. CWZ is a large regional hospital, with four nephrologists treating 630 patients in the outpatient renal clinics. Patients with CKD under nephrologists’ care who met the BR criteria were identified. These BR criteria were developed by nephrologists and GPs in a local consensus meeting^8^ and were based on (inter)national guidelines.^5–7^ Suitable patients were further informed about the study and asked to participate. Patients received an informed consent form from the nephrologists and were later seen at the renal nurses’ office. The nurse answered patients‘ questions, went through the treatment plan, took the signed consent forms, and distributed personal CKD information guides, the ‘kidney passport’ (in Dutch: ‘nierpas’). The kidney passport was developed in collaboration with the Dutch Kidney Foundation and includes general information, patients’ most recent measurements, blood results, medication, personal target values, and personal monitor frequency (see Supplementary Appendix S1). The aim of the kidney passport is to stimulate patient empowerment in treatment and follow-ups at the GP practice. The study’s setting was extended to the GP practice once the patients were referred back to primary care. The nephrologists sent extensive transfer letters to the patients’ GPs and remained available for consultation by telenephrology.^18,19^ Telenephrology is a web-based consultation system linked to the patient’s medical record. GPs open an online consultation form, and all data relevant to CKD is uploaded automatically. Inclusion took place from January to September 2019. The follow-up after the inclusion of patients was 1 year.

### Study population

Patients with CKD under nephrologists’ care who met the BR criteria were included.

The BR criteria were defined as follows:

- patients with stable kidney function, blood pressure, and metabolic parameters, where renal replacement therapy was not expected within 5 years (see Box 1);
- patients in whom patient and nephrologist concluded that renal replacement therapy did not contribute to quality of life or life expectancy; and
- patients with glomerular haematuria and/or proteinuria with stable kidney function, proteinuria <1 g, and stable blood pressure, where follow-ups were guaranteed to conform with nephrologists recommendations (exception: immunoglobulin A nephropathy with proteinuria >0.5 g, which could be a reason to start immunosuppressive drugs).

Box 1 **Definition of stable kidney function, blood pressure, and metabolic parameters**

Stable kidney function:

1a) Decrease of estimated glomerular filtration rate (eGFR) <25% compared with the first measurement within 5 years without a decrease in chronic kidney disease (CKD) stage

OR

1b) Decrease of eGFR <5 ml/min/1.73 m^2^/year, determined with at least three measurements

AND

2) <0.5–1.0 g proteinuria in a 24-hour urine test (depending on the patient’s kidney pathology: in case of glomerular haematuria with proteinuria <0.5 g, in case of vascular pathology <1.0 g).

Stable blood pressure:

Patients with blood pressure at or below their individual target value and which remained stable during two consultations at the outpatient department; no drug adjustments were necessary or possible, and no new drugs were started.

Stable metabolic parameters:

Patients who did not need any adjustment of medication targeting phosphate, calcium, parathormone, or haemoglobin during the last two consultations.

Exclusion criteria were as follows:

- rapidly decreasing kidney function expressed by a drop in eGFR >5 ml/min/1.73 m^2^/year or a decrease of >25% in 5 years or proteinuria >1 g;
- eGFR <30 ml/min/1.73 m^2^, with progression and who were suitable candidates for renal replacement therapy;
- expectation of requiring renal replacement therapy within 5 years (current age taken into consideration and assuming the worst-case scenario);
- use of immunosuppressive drugs relating to a renal disease;
- polycystic kidney disease at aged <50 years;
- glomerular haematuria with proteinuria >0.5 g, which could be a reason to start immunosuppressive drugs; and
- multiple morbidities or metabolic complications that could not be regulated in primary care, or medications shifts within the last three consultations.

### Data collection and analyses

Each participant’s data were collected twice. First, from the hospital information system directly after inclusion (T0). The following were recorded: demographics; most recent blood and urine results; blood pressure measurements; personal target values; and medication. The transfer information letter intended for the GP was taken. eGFR was extracted using the Chronic Kidney Disease Epidemiology Collaboration (CKD-EPI) formula. Second, 1 year after each patient’s inclusion, GPs were asked for data on follow-up and actual medication from their medical records (T1). QoC indicators were derived from the Dutch CKD guidelines on monitoring of disease progression (eGFR and albumin-to-creatinine ratio [ACR] assessment), blood pressure measurements, and achievement of blood pressure targets.^6^ If nephrologists demanded the monitoring of metabolic parameters (assessment of potassium, haemoglobin, calcium, and phosphate), this was added as a QoC indicator. It was examined whether monitoring frequency conformed with nephrologists’ requests. The stability of kidney function, blood pressure, metabolic parameters, medication changes, and the number of telenephrology consultations after 1 year were assessed. The patients who remained in primary care were counted and nephrologists’ involvement during follow-up and reasons for re-referral from primary to secondary care were investigated. Also, satisfaction and suggestions concerning BR to primary care by patients and GPs were evaluated by questionnaire (see Supplementary Appendices S1 and S2). Participants completed the questionnaires in Dutch, all answers were translated by a native speaker. All qualitative data were examined by two researchers independently. The researchers discussed the data and reached a consensus on the themes that emerged. Data were stored using Castor, an authorised web-based system that enables researchers to build electronic case report forms and store data.^20^

## Results

### Demographics

In total, 19 patients agreed to be referred back to primary care. One patient preferred BR without participating in the study. Consequently, 18 patients participated: 10 males and eight females (see Table 1). The mean age was 73 years (standard deviation [SD] 6.3), with a range of 62–86 years. Average eGFR (CKD-EPI formula) was 33.2 ml/min/1.73 m^2^ (SD 5.8); mean ACR was 6.7 mg/mmol (SD 8.8); mean systolic blood pressure was 132 mmHg (SD 16.8), and mean diastolic blood pressure 71 mmHg (SD 8.4) at baseline. All patients had comorbidities besides CKD. In all patients, values of serum potassium, phosphate, calcium, and haemoglobin were within normal ranges.

**Table 1.** Demographics of patients before start of the pilot (T0)

Number	Sex	Age, years	Nephrological diagnosis	Kidney functionChronic Kidney Disease Epidemiology Collaboration , ml/min/1.73 m^2^	AlbuminuriaACR, mg/mmol	Blood pressure, mmHg	Comorbidity
**1**	M	70	Renovascular and postrenal problem	30	26.2	134/77	HypertensionProstate cancer*/Seminoma testisPulmonary embolismDepression
**2**	M	69	Glomerular disease	29	25.4	121/69	HypertensionHyperglycaemia
**3**	F	77	Diabetes mellitus	35	0.8	144/69	Hypothyroidism
**4**	F	66	Diabetes mellitus and TIN	30	1.9	160/79	Atrial fibrillationMitral insufficiency
**5**	F	70	Renovascular and drug-induced	28	0.6	121/63	HypertensionHypothyroidism
**6**	M	81	Renovascular	37	1.8	138/63	HypertensionHeart failureDiabetes mellitusPancreas insufficiency
**7**	M	74	Nephrectomy after incidentaloma	30	4.7	134/68	Diabetes mellitusAnaemia
**8**	M	86	Renovascular	28	16.7	127/73	Myocardial infarctionCVAAneurysm iliac artery
**9**	F	66	Renovascular	39	4.2	159/80	Hypertension
**10**	M	82	Unknown	26	3.0	117/73	HypertensionAnaemia
**11**	F	78	Kidney atrophy	30	4.1	90/50	CVALung cancerNon-Hodgkin lymphomaColon cancerLarynx cancerAorta valve stenosis
**12**	M	77	Renovascular	27	4.5	124/76	Diabetes mellitusHypertensionAngina pectorisCOPD GOLD IIAnaemiaAneurysm abdominal aortaBladder cancer
**13**	F	67	Renovascular and nephrectomy	31	0.0	116/61	Coronary artery diseasePeripheral vascular disease
**14**	M	76	Renovascular	32	0.2	127/69	Hypertension
**15**	M	71	Glomerular disease	42	3.8	144/83	Diabetes mellitusCoronary artery disease
**16**	F	72	Renovascular	36	19.7	138/66	HypertensionDiabetes mellitusPeripheral artery disease
**17**	F	62	TIN	41	1.2	148/73	HypertensionObesityTIAGoutOSASCholecystectomy
**18**	M	70	Unknown	46	1.0	140/83	TIA

ACR = albumin-to-creatinine ratio. CKD = chronic kidney disease. COPD = chronic obstructive pulmonary disease. CVA = cerebrovascular accident. F = female. GOLD = Global Initiative for Chronic Obstructive Lung Diseases. M = male. OSAS = obstructive sleep apnea syndrome. TIA = transient ischaemic attack. TIN = tubulointerstitial nephritis.

### Renal outcomes after 1 year of follow-up in primary care

After follow-up, data from two patients (p) were missing (p9 and p14): one GP did not respond to the letters; and one GP missed the letter about patient BR and did not monitor the patient. The other 16 patients had stable kidney function after 1 year of follow-up (see Supplementary Table S1, definitions of stability in Box S1). The mean eGFR at the end of the study year was 34.3 ml/min/1.73 m^2^ (SD 6.4) and the mean ACR was 4.1 mg/mmol (SD 2.6). Two patients’ blood pressure was too high; one received extra blood pressure medication.

All patients were treated and followed in primary care after 1 year. One patient (p1) called the outpatient clinic after the study for a follow-up; they were seen twice at their request and were referred back to primary care. In all patients, metabolic parameters were stable during follow-up.

### Quality of care in primary care

One patient did not receive blood pressure measurements during the follow-up period. Another lacked a proteinuria measurement during the follow-up. As mentioned, one patient was not monitored at all, and there was no medication change for one patient with high blood pressure. GPs decreased the monitoring frequency recommended by the nephrologist for six patients, and for three patients for no apparent reason. Reasons for adjusting monitoring frequency were as follows: many hospital admissions including control of kidney function; patient refusing to take blood samples because of COVID-19; and the GP missed the transfer letter (see Supplementary Table S1). Telenephrology was used once in nine cases: one GP had a question about albuminuria and another about blood pressure. All others used telenephrology for feedback for the nephrologists, as requested by the nephrologists in the transfer letter.

### Patients’ experiences

One patient did not respond to the letters. Three patients indicated that they did not want to complete the final evaluation without giving a reason. One patient died owing to COVID-19 just after the follow-up period, so was unable to fill in the evaluation. The survey sent to the patients concerned the following four areas: information provision; use of the kidney passport; monitoring by the GP; and the GP and nephrologist’s collaboration. For nominal results of patients’ answers, see Supplementary Table S2.

#### Information provision

Most patients (*n* = 10/13) were satisfied with the nephrologists’ explanation about the cause of their CKD. One patient said:

*‘Extensive explanation by the nephrologist, taking into account my medical background as a dentist.’ (P1)*

Participants remembered some important information, including *‘it has something to do with my blood pressure‘* (P9), *‘the kidney function must be above 30‘*, (P7) and *‘I must pay attention to medication use‘ (P4).*

Three patients were not satisfied with the nephrologists’ explanation. They did not remember the nephrologist mentioning the cause. One said:

*‘I haven’t really heard anything about the cause.’ (P14)*

Patients were satisfied with information about treatment. They received lifestyle advice, such as weight loss, smoking cessation, and decreasing protein intake. It was also clear that the GP’s task in this study would be *‘monitoring of kidney function‘* (P18)

#### Use of the kidney passport

Patients who used the kidney passport were enthusiastic, particularly about having insight into their own target values. One patient said:

*‘I am satisfied about the target values, I can check* [them] *myself.’ (P6)*

Four patients did not examine the kidney passport after BR to primary care. Their comments included, *‘I already know the information‘* (P5), *‘I lost it‘* (P9), and *‘I never received the kidney passport’* (P13).

#### Monitoring by the GP

Eight patients thought they had been monitored as instructed by the nephrologist. Five patients noticed differences in monitoring: one patient was not monitored at all, one patient was not weighed by the GP, and three patients mentioned being monitored less frequently then prescribed. Two patients experienced difficulties with going to the GP’s practice because of COVID-19:

*‘I think the check was too long ago, I would like to be checked more often.‘ (P17)*

*‘Because of COVID-19 I could not easily go to the doctor*.*‘ (P12)*

Over half (*n* = 8/13) of patients took initiative and approached their GPs themselves to continue monitoring. One patient said:

*‘Another check was necessary. I did not receive a call for this from the GP and I had to take the initiative myself. That was not a problem in my case, but it may be for someone else*.*’ (P1)*

Only one patient had no confidence in the GP’s treatment because of the lack of monitoring. Another patient indicated that they did not know whether to trust the GP:

*‘I would have liked to remain under the control of my nephrologist.’ (P1)*

Other comments from patients included, *‘I have a good empathic doctor‘* (P4) and *‘I have good contact with my GP. Everything was thoroughly discussed during the check-up. Everything can be discussed’* (P6).

#### Collaboration between GP and nephrologist

Seemingly, most patients were unaware of communication between the GP and the nephrologist. Only four patients knew about the GP’s telenephrology consultations. Patients were thus unable to value the collaboration.

### GPs’ experiences

The GPs filled in 17 evaluation forms. Patients were referred back to 15 different GPs. Two GPs received two patients who were referred back to their practice and completed an evaluation form for each patient. One GP did not respond to the letters. See Supplementary Table S3 for nominal results of the questionnaires completed by GPs.

#### Information provision

Two GPs did not read the referral back letter: one GP had taken over the practice of a retired GP and noticed the control of cardiovascular risk factors had not been started; and the other missed the letter completely. This last letter was received by a colleague from the same practice, who did not inform the GP. In three cases, the letter was unclear after digital transmission owing to a shift of personal target values in the table. All other GPs (*n* = 14) evaluated the letter as clear. One GP said:

*‘A very clear letter, a cookbook with strict directions; but I am not a servant or a physician assistant to be managed in this way*.*’ (GP of P18)*

#### Organisation of care

Ten GPs ensured active CKD monitoring after BR to primary care, mostly by instructing the practice nurse. Other GPs instructed the patient to call the practice every 3 months for a laboratory form and an appointment. GPs who did not actively ensure follow-up indicated that there was *‘too much work in my current practice‘* (GP of P3) or that is was the *‘patient’s responsibility‘* (GP of P1)

In seven practices, the patients were only seen by the GP; in six practices both GPs and practice nurses were involved; in three practices, the practice nurse was in charge of monitoring; and in one practice, no follow-up by the GP or the practice nurse occurred. GPs responded:

*‘Our practice nurse is not competent.’ (GP of P6)*

*‘The care is provided by the practice nurse as much as possible but given the non-compliance and bad lifestyle of the patient, monitoring often is conducted by the GP.’ (GP of P15)*

*‘The patient will automatically enter our cardiovascular care programme where kidney function assessment is included.’ (GP of P3)*

#### Knowledge and follow-up

Ten GPs said they executed the follow-up as prescribed by the nephrologist. Several reasons for adjusting the monitoring were mentioned:

*‘The patient has not given blood for testing since COVID-19*.*’ (GP of P2)*

*‘The patient has stable kidney function.’ (GP of P3)*

*‘Frequent emergency room and specialist visits in between.’ (GP of P4)*

*‘We did the lab checks, although three monthly monitoring seems quite lavish to me.‘ (GP of P18)*

Only two GPs reported a lack of knowledge when monitoring moderate-to-advanced patients with CKD. One GP indicated:

*‘I need further training, although I don’t know if I want to do that on top of all my other tasks.’ (GP of P17)*

CKD care substitution was mostly seen as not or slightly onerous:

*‘... not more onerous than an average complex patient. But I only have a few similar cases, so it is not in my system.’ (GP of P5)*

*‘It’s a bit onerous because I have to guard the patient to take blood samples.’ (GP of P2)*

*‘You need a practice nurse for all logistic matters, but we don’t have enough manpower for that.’ (GP of P11)*

One GP experienced the substitution of care as an increase of workload:

*‘It takes time to read what exactly is intended, and you have to read it again every three months, because this is not an everyday problem for a GP.’ (GP of P17)*

Other GPs reported various barriers during follow-ups, including *‘a patient with a very low motivation, not consistent with the number of check-ups‘* (GP of P15), *‘the study is onerous‘* (GP of P6)*,* and *‘apparently we had to use the kidney passport but we decided not to use it’* (GP of P18).

#### Use of the web-based consultation system (telenephrology) and the kidney passport

Two GPs used telenephrology because they needed to consult a nephrologist. One question was about blood pressure management, the other was about target values. All other uses of telenephrology were because consultations were requested as part of the study.

Most of the GPs (*n* = 11/17) did not use the kidney passport. GPs argued:

*‘I am not aware of the benefits of the kidney passport.’ (GP of p13)*

*‘The patients did not bring the kidney passport every visit.’ (GP of p15)*

*‘... too much administration.’ (GP of p3)*

*‘The patients filled in the kidney passport himself, I have the results in the computer.’ (GP of p1)*

## Discussion

### Summary

This pilot study showed BR is safe and feasible for patients with CKD who meet the BR criteria. Patients had good renal outcomes after 1 year. There would likely have been no differences in outcomes for patients between GP treatment and nephrologist treatment; however, there is room for improving QoC in primary care. Particularly monitoring frequency and completeness of monitoring could be improved, although this must be balanced against all other aspects of care. Follow-up frequency was decreased by several GPs for various reasons. Patients were generally satisfied with nephrologists’ information provision, the kidney passport, and treatment by the GP, but expected more frequent monitoring. GPs believed they had sufficient knowledge to treat patients with stable moderate-to-advanced CKD in primary care, but indicated they needed support besides a transfer letter. Some GPs did not trust aftercare to their practice nurses.

### Strengths and limitations

It is a strength that patients were involved and that their opinions and experiences were asked. Involving patients and stimulating self-management remains a challenge. A limitation of this feasibility study is its small sample size. The study was intended as an initial exploration of BR of patients with stable moderate-to-advanced CKD. Unfortunately, it was impossible to evaluate all participating patients since patients were free to leave the study at any time. The follow-up period was long (1 year), which induced a risk of loss to follow-up. GPs, who were not included in the study beforehand in the way patients were, were asked for reactions. Some GPs found participation very time-consuming. The extensive explanations for patients in outpatient clinics were also very time-consuming. For future BR procedures, a good explanation for patients and GPs about BR will take time.

### Comparison with existing literature

The rising pressure on hospital facilities and the ambition to provide care in patients’ own environments necessitate a critical consideration of the need for CKD hospital care. Accordingly, it is important to prevent referrals using telenephrology^19^ and to refer patients with stable CKD back to primary care when possible. A study from Wales showed patients with stable CKD who were discharged back to primary care were at low risk for disease progression. Those referred back to primary care because of failing to attend renal clinics were at risk of deteriorating renal function.^21^ Another study showed nearly 30% of patients with moderate-to-advanced CKD could be treated in a shared care construction. These patients had good outcomes concerning risk of death and renal replacement therapy compared with patients solely treated in hospital.^22^ Both in the present study and the two previously mentioned studies, the median age was relatively high. This reflects the fact that most patients had renovascular of diabetic aetiology and are more likely to have stable kidney damage. Very limited research has been done concerning BR in nephrology, but the principle and quality of monitoring in primary care have been researched in other specialties, for example, heart failure in cardiology.^23^ This study also indicates that some of the outpatient population could be monitored by GPs.^24^

Most GPs in the present study felt they were capable of taking care of these patients with CKD. They experienced no differences with other complex patients treated in primary care; however, most quantitative studies have shown educational gaps and even unawareness of CKD in GPs.^25^ Facilitators for CKD care in primary care are access to the nephrologist^26^ and a proactive approach, where multimorbid conditions are treated together.^25,27^

A downside of this substitution of care from different specialisms to the GP is the increase in GP workload. This has not been well researched yet, but some GPs in the present study reported an extensive workload because of the BR. A study into the BR of patients with chronic cancer indicated a workload limited increase for GPs, since most patients already have comorbidities.^28^ These comorbidities need to be periodically checked by the GP anyway, with some overlapping content. It is common knowledge that consultation time and workload increase with the number of chronic conditions.^29^ In the present study population, all patients had comorbidities, such as hypertension and diabetes. In the Netherlands, patients with chronic conditions like these are enrolled in specific disease-management programmes, where they are regularly checked by a nurse practitioner and by the GP once a year.^30^

The present study shows patients are not aware of collaboration between GPs and nephrologists. This is in line with the findings of another study, in which patients considered collaboration between GP and specialist as important and self-evident, but did not know what it entails.^31^ If patients know that their case is being discussed with a nephrologist, this may increase confidence in treatment and GP. A likely mode for improvement is likely giving patients insight into written communications between GPs and nephrologists, with a shared medical file, for example. The kidney passport alone is insufficient.

### Implications for practice and research

Nephrologists’ main concern is whether patients will be monitored adequately when referred back to primary care.^9^ Nephrologists may be more likely to refer patients back to primary care if they know that follow-up is guaranteed. In one-third of the cases, GPs adjusted the monitoring frequency — recommended by the nephrologist — downwards. GPs also deviated from guidelines in this regard, since guidelines recommend monitoring frequencies for each different CKD stage. It is unclear whether monitoring frequency was discussed with the patients or whether patients influenced monitoring frequency. The importance of frequent monitoring must be brought to the attention of GPs. Ensuring follow-up is not easy since GPs’ knowledge and visions, and GP practice organisations, vary greatly. In the Netherlands, it is not required to keep separate CKD registers in practices. It is GPs’ responsibility to code CKD in the patient’s medical file with an International Classification of Primary Care code and to periodically report the current eGFR to the pharmacist. Follow-up could be established by patient participation, IT solutions, or monitoring in a disease-management programme, besides guidelines and transfer letters.

Engaging patients with only personalised information guides proved to be insufficient. Patients were not aware of cooperation between GPs and nephrologists, so more interventions are needed to give patients greater insight. These interventions could include a shared digital protected web-based platform containing laboratory results, blood pressure, and written consultations, accessible by the nephrologist, GP, and patient.^32^ Good agreements must then be made about who bears what responsibilities, since unclear roles are perceived as a barrier to collaboration.^26^

Several participating GPs suggested that even if the patients who were back referred with CKD had no other comorbidities, they could be followed in the prevailing disease-management programmes in primary care. One condition for organising this chronic care in disease-management programmes is that follow-up is well described by nephrologists. A practice nurse could be deployed to guarantee monitoring and signal deviations from the patient‘s target values. The nephrologist can be consulted in case of progression or metabolic dysregulation. Rapid communication from GPs with nephrologists could be achieved by shared digital files. Allowing nephrologists to examine shared files also addresses one of the other contradictions that affects GPs: the need for structure and embedding CKD care versus the experience of being patronised by the nephrologist.

The implementation of BR criteria and the BR of patients with stable moderate-to-advanced CKD can now be studied on a larger scale, and in multiple hospitals and GP practices. Appropriate IT and healthcare apps need to be studied to offer patients more insight in treatment and communication, and to give more self-management options. Research into BRs’ sustainability and cost-effectiveness is a crucial next step.

In conclusion, BR seems safe and feasible for patients with CKD with stable moderate-to-advanced stable CKD who meet the correct criteria. Patients have good renal outcomes after 1 year of follow-up in primary care. QoC at GP practices can be improved, particularly monitoring frequency, which was decreased by GPs for several reasons. Generally, patients are satisfied with treatment by GPs and the kidney passport. Overall, GPs believed that they have sufficient knowledge to treat patients with stable moderate-to-advanced CKD in primary care, but indicated that they need support besides a transfer letter. Sufficient follow-up in primary care must be ensured by more interventions than a transfer letter and engaging patients with kidney passports.
